# Genetic Analysis of *Citrobacter* sp.86 Reveals Involvement of Corrinoids in Chlordecone and Lindane Biotransformations

**DOI:** 10.3389/fmicb.2020.590061

**Published:** 2020-11-09

**Authors:** Agnès Barbance, Oriane Della-Negra, Sébastien Chaussonnerie, Valérie Delmas, Delphine Muselet, Edgardo Ugarte, Pierre-Loïc Saaidi, Jean Weissenbach, Cécile Fischer, Denis Le Paslier, Nuria Fonknechten

**Affiliations:** ^1^Génomique Métabolique, Genoscope, Institut François Jacob, CEA, CNRS, Univ Evry, Université Paris-Saclay, Evry, France; ^2^Laboratoire de Cancérologie Expérimentale, IRCM, Institut François Jacob, CEA, Université Paris-Saclay, Fontenay aux Roses, France

**Keywords:** chlordecone, lindane, dechlorination, corrinoid, cobalamin, *Citrobacter*, gene deletion, degradation

## Abstract

Chlordecone (Kepone®) and γ-hexachlorocyclohexane (γ-HCH or lindane) have been used for decades in the French West Indies (FWI) resulting in long-term soil and water pollution. In a previous work, we have identified a new *Citrobacter* species (sp.86) that is able to transform chlordecone into numerous products under anaerobic conditions. No homologs to known reductive dehalogenases or other candidate genes were found in the genome sequence of *Citrobacter* sp.86. However, a complete anaerobic pathway for cobalamin biosynthesis was identified. In this study, we investigated whether cobalamin or intermediates of cobalamin biosynthesis was required for chlordecone microbiological transformation. For this purpose, we constructed a set of four *Citrobacter* sp.86 mutant strains defective in several genes belonging to the anaerobic cobalamin biosynthesis pathway. We monitored chlordecone and its transformation products (TPs) during long-term incubation in liquid cultures under anaerobic conditions. Chlordecone TPs were detected in the case of cobalamin-producing *Citrobacter* sp.86 wild-type strain but also in the case of mutants able to produce corrinoids devoid of lower ligand. In contrast, mutants unable to insert the cobalt atom in precorrin-2 did not induce any transformation of chlordecone. In addition, it was found that lindane, previously shown to be anaerobically transformed by *Citrobacter freundii* without evidence of a mechanism, was also degraded in the presence of the wild-type strain of *Citrobacter* sp.86. The lindane degradation abilities of the various *Citrobacter* sp.86 mutant strains paralleled chlordecone transformation. The present study shows the involvement of cobalt-containing corrinoids in the microbial degradation of chlorinated compounds with different chemical structures. Their increased production in contaminated environments could accelerate the decontamination processes.

## Introduction

Chlordecone is a toxic organochlorine persistent organic pollutant (POP) included in the Stockholm Convention in 2009 (Jennings and Li, 2015). It has been manufactured for several years in the United States (Epstein, 1978) until it was banned in 1975. Its production at the Hopewell plant (Virginia) led to acute exposure of workers and a massive pollution of the James River and its surroundings, which extended more than 100 miles toward the Chesapeake Bay (Dawson et al., 1979; Huggett and Bender, 1980). Since then, the contamination has been slowly declining, due to chlordecone burying over the time into riverbed sediments (Trotman and Nichols, 1978; Nichols and Cutshall, 1981; Nichols, 1990; Unger and Vadas, 2017). In banana plantations of the French West Indies (FWI, Guadeloupe and Martinique Islands), chlordecone usage for its insecticide properties against the banana black weevils lasted from 1972 until 1993 (Vilardebo et al., 1974; Le Déaut and Procaccia, 2009; Lesueur-Jannoyer et al., 2016), resulting in long-term pollution of environmental compartments and the local food chain (Lesueur-Jannoyer et al., 2016). Acute and chronic exposure to chlordecone was the cause of human health harm such as increased risk of prostate cancer, motor and cognitive development disorders in young children, premature births (Epstein, 1978; Multigner et al., 2018; Maudouit and Rochoy, 2019), and subsequent socio-economic issues for the FWI and James River areas (Lesueur-Jannoyer et al., 2016; Unger and Vadas, 2017).

**Figure 1:**
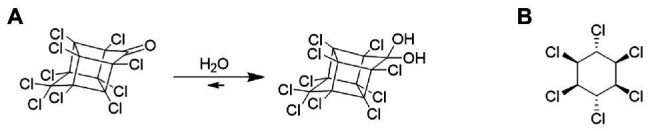
**(A)** Chemical structure of chlordecone mostly present under its hydrated form Wilson and Zehr (1978). **(B)** Chemical structure of γ-hexachlorocyclohexane (lindane).

Due to its specific chemical structure (a bis-homocubane cage) and its perchlorinated nature, chlordecone was considered as non-degradable in the environment for a long time (Epstein, 1978; Cabidoche et al., 2009), despite some laboratory-based evidences of transformation (Figure 1). Two studies suggested possible aerobic degradation of chlordecone (Orndorff and Colwell, 1980; George and Claxton, 1988). And later on, the conversion of chlordecone into unknown polar and nonpolar transformation products (TPs) in the presence of the anaerobic Archaeon *Methanosarcina thermophila* was reported (Jablonski et al., 1996). In 2011, the bacterium *Pseudonocardia* sp. KSF27 isolated from endosulfan-contaminated soils was also reported as transforming chlordecone in aerobiosis (Sakakibara et al., 2011). A very low level of chlordecone mineralization was also observed in a microcosm incubated during several months in aerobic conditions (Fernández-Bayo et al., 2013). However, no TP was described to confirm the apparent degradation in these two studies. More recently, we showed, that under anaerobic laboratory conditions, bacterial consortia and isolated bacteria (*Citrobacter* sp.86 and *Desulfovibrio* sp.86) could transform chlordecone into numerous TPs of different types (Figure S1; Chaussonnerie et al., 2016; Della-Negra et al., 2020). In addition, we detected the same TPs in environmental samples from Martinique Island, suggesting that a similar natural degradation occurs in Martinique soils (Chevallier et al., 2019; Della-Negra et al., 2020). This phenomenon was independently confirmed by others with the detection of chlordecone TPs in Guadeloupe soils (Lomheim et al., 2020). However, to date, the mechanisms of chlordecone degradation remain unknown and do not seem to be mediated by organohalide-respiring bacteria.

*Citrobacter* sp.86, related to *Citrobacter amalonaticus*, has been isolated from a chlordecone-degrading bacterial consortium and its genome was sequenced (Chaussonnerie et al., 2016). This facultative anaerobic bacterium was demonstrated to fully transform chlordecone anaerobically. The chlordecone TPs produced by *Citrobacter* sp.86 were classified into three families: A (hydrochlordecones) resulting from dechlorination, B (polychloroindenes) arising from the opening of the bis-homocubane cage, dechlorination, and the loss of one carbon atom and two oxygen atoms, and C (polychloroindenecarboxylic acids) obtained from chlordecone ring-opening dechlorination (Chevallier et al., 2019; Figure S1). In the previously described microbiological conditions, TP B1 (2,4,5,6,7-pentachloroindene) was found predominant according to Gas Chromatography coupled to Mass Spectrometry (GC-MS) analysis. Similar TPs, belonging to families A and B, were previously obtained by chemical degradation of chlordecone in the presence of vitamin B_12_ (Schrauzer and Katz, 1978; Ranguin et al., 2017), while hydrochlordecones (family A) were the only products in presence of zero-valent iron (Belghit et al., 2015). Combining vitamin B_12_ and a reducing agent generated practically a similar pattern of chlordecone TPs as those observed in *Citrobacter* sp.86 or *Desulfovibrio* sp.86 anaerobic cultures (Chevallier et al., 2019; Della-Negra et al., 2020). However, comparison of the carbon isotope fractionation of chlordecone in microbiological degradation using *Citrobacter* sp.86 and in chemical degradation mediated by vitamin B_12_ suggested different mechanistic pathways (Chevallier et al., 2018).

Genome sequence analysis of *Citrobacter* sp.86 revealed that this facultative anaerobic bacterium harbors the complete anaerobic cobalamin synthesis pathway (Chaussonnerie et al., 2016). As none of the key enzymes involved in organohalide-respiration (reductive dehalogenases) were detected in the *Citrobacter* sp.86 genome sequence (van der Ploeg et al., 1991; Nagata et al., 2007; Fincker and Spormann, 2017), it was hypothesized that cobalamin and/or other corrinoids might be involved. To investigate this hypothesis, knockout mutants of *Citrobacter* sp.86 were constructed in which cobalamin synthesis was impaired. These mutant strains, that had one or more genes deleted, were tested for their ability to promote chlordecone degradation, in comparison with the *Citrobacter* sp.86 wild-type.

More than 40 years ago, another *Citrobacter* species (*Citrobacter freundii*) was described to anaerobically degrade a different chlorinated pesticide: lindane (Figure 1), also known as γ-hexachlorocyclohexane, γ-HCH (Jagnow et al., 1977). As for the transformation of chlordecone by *Citrobacter* sp.86, this transformation seemed to be co-metabolic. Lindane, toxic for humans and other organisms, has been widely used over the last 7 decades for agricultural, veterinary, and even human sanitary usages as a pesticide, wood protector, or against scabies and lice, generating millions of tons of waste over a wide range of countries (Middeldorp et al., 1996; Lal et al., 2010; Álvarez et al., 2012; Vijgen et al., 2019; Wacławek et al., 2019). The hydrophobicity and chemical stability of lindane, with half-life in soil and water spanning years, its ongoing use in some countries as well as the existence of stockpiles also designate it as a legacy organochlorine compound; and it is still detected in environmental compartments (Laquitaine et al., 2016; Saez et al., 2017; Vijgen et al., 2019). Lindane was also frequently used in the FWI before being largely replaced by chlordecone, and ultimately banned in 2009 due to its recalcitrance to degradation and toxicity (Nolan et al., 2012; Dereumeaux et al., 2019).

Different bacterial consortia or single bacteria isolated from soils, sediments, plants, or farm fields have been described for their ability to degrade lindane aerobically (Lal et al., 2006, 2010; Saez et al., 2017; Zhang et al., 2020). Under these conditions, the aerobic degradation relies on the presence of the *lin*A-E genes, which encode among others a dehydrochlorinase and a haloalkane dehalogenase providing chlorinated benzene derivatives followed by the *lin*F-J genes that ultimately fuel them up into the central metabolism (Senoo and Wada, 1989; Kumari et al., 2002; Dogra et al., 2004; Böltner et al., 2005; Endo et al., 2005; Lal et al., 2010; Cuozzo et al., 2017). Lindane partial degradation has also been observed in some Guadeloupean soils where it was suggested that lindane aerobic degradation could occur, probably through bacteria closely related to the family *Sphingomonadaceae* (Laquitaine et al., 2016).

*Citrobacter* sp.86 is a facultative anaerobe, but its genome does not encode homologs of the *lin* genes. Co-metabolic anaerobic degradation of lindane was reported for facultative anaerobic bacteria like *C. freundii* (Jagnow et al., 1977) but also for strict anaerobes like a *Clostridium* sp., *Desulfovibrio gigas* and *Desulfococcus multivorans* (Macrae et al., 1969; Ohisa and Yamaguchi, 1978; Boyle et al., 1999; Badea et al., 2009; Mehboob et al., 2013), in bacterial consortia (Qiao et al., 2020; Zhang et al., 2020) and finally using slurry systems (Quintero et al., 2005; Robles-González et al., 2008; Camacho-Pérez et al., 2012). In 2011, an anaerobic enrichment culture was showed to use lindane as electron acceptor; however, no organohalide-respiring bacteria were detected in the consortium (Elango et al., 2011). In 2018, two *Dehalococcoides mccartyi* strains (195 and BTF08) were also found to use lindane as an electron acceptor (Bashir et al., 2018). In contrary to the aerobic degradation pathway of lindane, two mechanisms were proposed in anaerobiosis. The first one involved two successive dichloroeliminations followed by a dehydrochlorination leading to chlorobenzene. The second one passes through a pentachlorocyclohexene intermediate to finally form 1,2-dichlorobenzene and 1,3-dichlorobenzene (Figure S1). Benzene was also observed in several conditions (Zhang et al., 2020).

However, to date, no genes or enzymes were found to be involved in these dechlorination processes (Lal et al., 2010; Zhang et al., 2020). The main goal of the present study was to elucidate whether the corrinoids produced by *Citrobacter* sp.86 play a role in the chlordecone and lindane biotransformations.

## Materials and Methods

### Chemicals

Chlordecone was obtained from Azur Isotopes (purity 98%). Chemical products used for microbiological media, vitamin B_12_ (>98%), chloro(pyridine)cobaloxime(III), lindane (97%), 1,3-dichlorobenzene (98%), 1,4-dichlorobenzene (99%), chlorobenzene (99.8%), and benzene (99.8%) were obtained from Sigma Aldrich. Titanium (III) citrate was prepared from titanium (III) chloride (>12% in HCl; Sigma Aldrich) and sodium citrate and neutralized with Na_2_CO_3_ (Chevallier et al., 2019). Dichloromethane (HPLC grade) was obtained from Fisher Chemical.

### Construction of *Citrobacter* sp.86 Knockout Mutant Strains

Knockout mutant strains in *Citrobacter* sp.86 were constructed using the λ-red recombinase technique as developed for *Escherichia coli* (Datsenko and Wanner, 2000) with some modifications. Briefly, a PCR product was generated by using primers with 50-nt 5'-extensions that are homologous to regions adjacent to the gene to be inactivated and 20-nt 3'-extremities hybridizing to the antibiotic resistance cassette of the plasmid pKD3 (chloramphenicol resistance cassette) or pKD4 (kanamycin resistance cassette). The amplicon was purified (QIAquick PCR Purification Kit, Qiagen) and introduced by transformation in electrocompetent *Citrobacter* sp.86 cells which harbor the helper plasmid pKD46-Gm (Doublet et al., 2008; kindly provided by Benoit Doublet). The thermosensitive plasmid pKD46-Gm contains the Red recombinase genes located under an L-arabinose inducible promoter and the gentamicin resistance cassette. Recombinant cells were selected on LB-agar plates containing the appropriate antibiotic (kanamycin or chloramphenicol, 50 μg/ml). The effective replacement of the target gene with the resistance cassette was verified by PCR-amplifying the targeted chromosomal locus of wild-type strain and of the recombinant candidates using specific primers located 200 nt upstream and downstream of the target gene and analyzing the PCR products by electrophoresis on agarose gels. The primer sequences used in this study are given in Tables S1, S2, and the designation and genotype of all bacterial strains as well as plasmids used in this study are given in Tables S3, S4.

### Culture Conditions

*Citrobacter* sp.86 strains were kept frozen at −80°C as stock glycerol (15%). Before starting phenotyping or degradation experiments, the bacteria were first grown aerobically at 37°C on LB plates with 100 μg/ml carbenicillin (the *Citrobacter* sp.86 genome encodes a beta-lactamase), and isolated colonies were used as inoculum.

For analysis of growth requirements (phenotyping), the *Citrobacter* sp.86 wild-type and mutant colonies issued from LB agar plates were then spread onto agar plates containing mineral medium MM previously described (Chaussonnerie et al., 2016) but without vitamin B_12_, supplemented with glucose (20 mM) or pyruvate (40 mM). When indicated, vitamin B_12_ and L-methionine were added at a final concentration of 0.3 μM or 0.3 mM, respectively. Plates were incubated aerobically at 37°C. For anaerobic assays, colonies issued from aerobic MM plates supplemented with methionine but without vitamin B_12_ were spread on MM plates containing Na_2_S (0.4 g/L), glucose (20 mM), or pyruvate (40 mM), with or without vitamin B_12_, and incubated at room temperature (rt) in a glove box (Unilab mBraun), under an N_2_/H_2_ (98/2; V/V) atmosphere.

Anoxic microbial incubations (degradation experiments) were performed in the glove box in daylight. Microbial liquid cultures were carried out in the mineral medium MM, but without vitamin B_12_, supplemented with pyruvate (40 mM) as carbon source, 2 g/L yeast extract, and 2 g/L tryptone. This complemented mineral medium was named MMpyt. The reductant was Na_2_S (0.4 g/L), and 0.1% of resazurin was added as an indicator of anaerobiosis. When indicated, it was adjusted at a pH differing from the standard (pH 7.0) by varying the proportions of the buffering system (KH_2_PO_4_/K_2_HPO_4_). Chlordecone and lindane were solubilized in dimethylformamide to a 200 mg/ml stock solution and used at 40 μg/ml or 20 μg/ml, respectively.

After growth on LB plates, a colony of each *Citrobacter* sp.86 strain resuspended in 50 μl NaCl 0.8% was transferred in the anaerobic glove box (Figure S2) and used for initial inoculation of a 2 ml culture in MMpyt with 100 μg/ml carbenicillin (culture C1). After 24 h, 50 μl of C1 was used to inoculate a 5 ml culture in MMpyt with 100 μg/ml carbenicillin and 10 μg/ml chlordecone or 10 μg/ml lindane (culture C2). After 24 h, C2 was finally used to inoculate a 50 ml culture in MMpyt and 40 μg/ml chlordecone or 20 μg/ml lindane (culture C3, contained in 100 ml glass serum vials), which was incubated for 4 months and 28 days for chlordecone and lindane, respectively. Each degradation experiment was done in duplicate. All experiments were monitored over time using GC-MS techniques. A negative control vial (without bacteria) was added to each tested condition.

### Organochlorines Sampling/Extraction for Chlordecone or Lindane Microbiological Culture Monitoring

Chlordecone monitoring was performed using GC-MS. After homogenization of the liquid cultures, 500 μl were collected and extracted twice using 250 μl isooctane. The combined organic layers were then analyzed through GC-MS analysis *via* liquid injection.

For lindane monitoring Headspace GC-MS (HS-GC-MS) was required. In this case, 700 μl of culture were sampled and put into a Chromacol 10-HSV vial of 10 ml (Agilent). The headspace gas was then analyzed through the Headspace tool (see below).

### Analytics

Gas Chromatography coupled to Mass Spectrometry analyses were used for chlordecone degradation monitoring and were carried out using a Thermo Fisher Focus GC coupled to a single-quadrupole mass spectrometer (Thermo Fisher DSQ II). The instrument was equipped with a non-polar 30 m × 0.25 mm × 0.25 μm DB-5MS column (Agilent J&W) and a split/splitless injector. Ionization conditions and the GC program have been described elsewhere (Chevallier et al., 2019).

Gas Chromatography Mass Spectrometry coupled with a Headspace trap (HS-GC-MS) analyses were used for lindane degradation monitoring and were performed on a Thermo Fisher Trace 1300 coupled to an ISQ 7000 VPI single quadrupole mass spectrometer. The instrument was equipped with a 30 m × 0.25 mm × 0.25 μm DB-624-UI column (Agilent J&W), a split/splitless injector, and an automatic sampler TriPlus RSH coupled to a HeadSpace tool. For MS analyses, the following standard working conditions were applied: electronic impact ionization, positive mode detection, ion source at 220°C, detector voltage 70 eV, and full scan mode m/z 33–300 (scan time 0.20 s). Injection and transfer line temperatures were set up at 200 and 280°C, respectively. Monitoring vials were incubated for 5 min at 50°C and sampled with a syringe at 50°C. One milliliter of the headspace gas was injected each time at a filling speed of 10 ml/min, an injection speed of 10 ml/min, and a penetration speed of 10 ml/s. The splitless injection mode was applied at 150°C. Carrier gas was helium at a constant flow rate of 0.5 ml/min. The GC program started at 30°C (hold time 6 min), continued with 15°C/min to 130°C (hold time 0.5 min), followed by 7°C min^−1^ to 250°C (hold time 10 min).

### Chemical Transformation of Chlordecone With Vitamin B_12_ or Cobaloxime

According to the protocol described elsewhere (Chevallier et al., 2019), to a solution of chlordecone (5.0 mg, 9.9 10^−6^mol, and 1 eq.) and vitamin B_12_ (4.1 mg, 3.0 10^−6^mol, and 0.3 eq.) or cobaloxime (1.2 mg, 3.0 10^−6^mol, and 0.3 eq.) in degassed water (30 ml) was added titanium (III) citrate basified to pH 12 with NaOH (3 M; 5 ml, 3.3 10^−4^mol, and 32 eq.). The reaction mixtures were stirred under N_2_ atmosphere at rt for 2 h; and monitored by GC-MS.

## Results and Discussion

### Strategy for the Targeted Deletion of Cobalamin Biosynthesis Genes in *Citrobacter* sp.86

**Figure 2:**
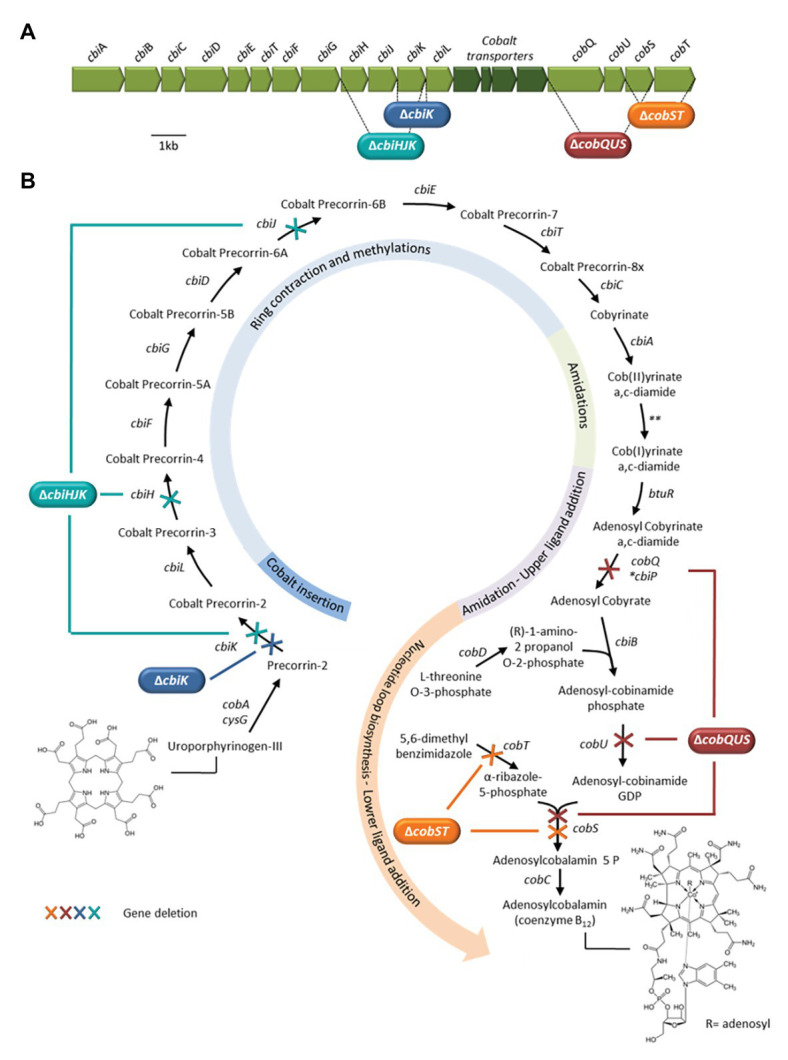
**(A)** Genetic organization of the main *Citrobacter* sp.86 anaerobic cobalamin biosynthesis gene cluster. The regions targeted for the deletion of one to three consecutive genes and replacement with an antibiotic resistance marker gene − see section the Materials and Methods − are shown. **(B)** Anaerobic cobalamin biosynthesis pathway in *Citrobacter* sp.86 based on genome annotation (^*^cbiP, synonym name for *cobQ*). Reactions blocked in the mutant strains are shown by crosses. ^**^In the anaerobic pathway, there is no dedicated enzyme for cobalt reduction (Fonseca and Escalante-Semerena, 2000).

Since most genes predicted to operate in a cobalamin biosynthesis pathway are clustered (Figure 2A), we chose to construct a test-set of mutant strains by targeted deletion of genomic coding regions encompassing multiple genes. In fact, these multiple gene deletions would certainly result in a defective cobalamin. Three mutant types were designed affecting different steps of the cobalamin molecule construction: (i) the insertion of the cobalt atom into the corrin ring and corrin core functionalization, (ii) the corrin core functionalization and nucleotide loop biosynthesis, and (iii) the nucleotide loop biosynthesis including the lower ligand insertion. They were respectively obtained by deleting the following gene combinations: *cbiHJK*, *cobQUS*, and *cobST*. In addition, based on preliminary results obtained for the *Citrobacter* sp. 86 *ΔcbiHJK* strain, a fourth mutant type *cbiK* affecting the cobalt insertion was constructed (Figure 2B). Detailed reactions catalyzed by proteins encoded by these genes are shown in Figure S3.

### Knockout Mutant Phenotypes

There are two methionine synthases in *Citrobacter* sp.86, one cobalamin-independent encoded by the gene *metE* and the other cobalamin-dependent, encoded by the gene *metH*. On a Δ*metE* background, a deletion that would impact the biosynthesis of cobalamin in *Citrobacter* sp.86 would have an effect on its viability. In a first step, we decided to test our constructions on this background.

Prototrophy of a *metE* knockout mutant strain in mineral medium and in the absence of any nutritional supplement other than a carbon source (glucose or pyruvate) was used as a physiological phenotypic indicator of the cellular cobalamin production. In the four Δ*metE* and Δ*cob/cbi* double mutant strains, we hypothesized that rescue of methionine auxotrophy would be achieved by exogenous cobalamin.

The growth phenotype of all strains was tested on solid rich or mineral medium under both aerobic and anaerobic conditions. Independently, all genotypes were also checked by PCR (Figure S4A).

As shown in Table S5, no growth defects were observed for any strain on LB rich medium, but growth phenotypes were more contrasted on mineral medium (Table S5; Figures S4B,C). The *Citrobacter* sp.86 Δ*metE* strain did not grow under aerobic conditions and this defect was alleviated by supplying exogenous cobalamin, which is required as a cofactor for MetH. In contrast, it grew under anaerobic conditions in the absence of any nutritional supplement indicating successful endogenous cobalamin biosynthesis. So, we concluded that *Citrobacter* sp.86 was unable to synthesize cobalamin under aerobic conditions. These results were consistent with the annotation of the genes involved in the anaerobic cobalamin pathway. Similarly, the enteric bacterium *Salmonella typhimurium* also synthesizes cobalamin *de novo* only under anaerobic growth conditions (Jeter et al., 1984).

The four *Citrobacter* sp.86 double mutant (Δ*metE* and Δ*cob/cbi*) strains were unable to grow on mineral medium under anaerobiosis, indicating that incomplete corrinoids cannot functionally replace the cobalamin cofactor for MetH functionality. Under these conditions, exogenous cobalamin fulfills this requirement. These results confirm the impairment of the cobalamin biosynthetic pathway in all *cob/cbi* knockout mutant strains made in this study.

In a second step, we also constructed four Δ*cob* or Δ*cbi Citrobacter* sp.86 knockout mutant strains to test for their ability to degrade chlordecone and lindane compounds. Based on the results of genotype verification by PCR and the physiological consequences of the cobalamin biosynthesis pathway disruption described above, these strains are impaired in the cobalamin synthesis pathway. None of these deletions impacts growth of *Citrobacter* sp.86 on mineral medium in the absence of cobalamin (Table S5; Figure S5; Supplementary Material), indicating that production of incomplete non-functional corrinoids was not harmful for the bacteria. Incubation of all these strains with chlordecone or lindane did not modify their growth phenotype (Figure S5; Supplementary Material).

### Chlordecone Transformation by *Citrobacter* sp.86 Wild-Type and Mutant Strains

**Figure 3:**
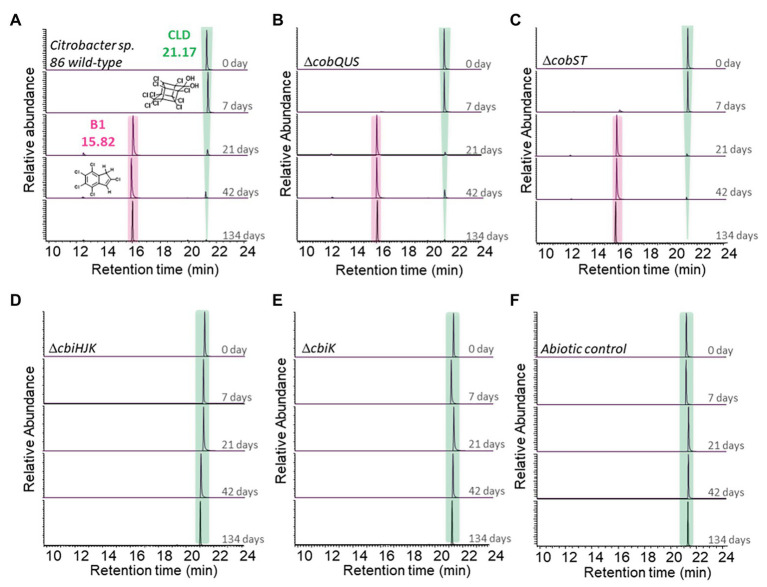
Transformation of chlordecone in *Citrobacter* sp.86 wild-type and mutant strain cultures over the time. GC-MS chromatograms (full scan mode) of extracted cultures at selected times incubated with **(A)** *Citrobacter* sp.86 wild-type, **(B)** Δ*cobQUS* mutant strain, **(C)** Δ*cobST* mutant strain, **(D)** Δ*cbiHJK* mutant strain, **(E)** Δ*cbiK* mutant strain, and **(F)** without bacteria (abiotic control). Numeric data of peak areas are available in Supplementary Material. The various mutant strains and the negative control (abiotic control) were incubated under the same conditions. For more clarity, a single chromatogram is displayed among the duplicates for each selected time.

The wild-type *Citrobacter* sp.86 and the test-set of cobalamin biosynthesis pathway mutant strains were incubated with chlordecone in MMpyt medium. Under these laboratory conditions, the wild-type strain reached the stationary-phase after 10 h. In reported bacterial transformations of chlordecone (Chevallier et al., 2019), the appearance of A and B TP families (monitored using GC-MS) systematically went along with C family (detected using LC-MS) while chlordecone disappearance was completed after several months. Here, we only focused on the detection of A and B families using GC-MS analysis. Transformation profiles of chlordecone in cultures were monitored regularly by GC-MS over a 4-month period. Interestingly, *Citrobacter* sp.86 and the mutant strains Δ*cobQUS* and Δ*cobST* showed the same chlordecone transformation profile (Figure 3; Supplementary Material): upon chlordecone incubation, trace amounts of B1 were detected on the 7th day. After 21 days, B1 was the main chlordecone TP detected using GC-MS analysis, as expected (Chaussonnerie et al., 2016; Chevallier et al., 2018, 2019). The transformation did not occur while the cells were actively growing but rather mostly after entry into the lysis phase (Figure S5). On the other hand, *Citrobacter* sp.86 Δ*cbiHJK* and Δ*cbiK* did not transform chlordecone even after an additional extensive period of incubation (a total period of 201 days, Supplementary Material), just as in the negative control without bacteria. In the light of these results, it can be assumed that suppression of the corrinoid lower ligand or modifications of the corrin core patterns do not affect the ability of *Citrobacter* sp.86 to degrade chlordecone. In contrast, inactivating the cobalt insertion step prevents chlordecone degradation, confirming the involvement of cobalamin or other corrinoids in chlordecone transformation. However, the corrinoids, which are functional for the chlordecone degradation process, are not necessarily complete cobalamin in contrast to the functional cofactor involved in enzymatic reactions. All these results are consistent with a transformation of chlordecone by a non-enzymatic process involving corrinoids with an inserted cobalt atom. So far, no mechanism can assess how cobalamin would react with chlordecone. However, assuming that chlordecone transformation would be the result of the action of corrinoids released by *Citrobacter* sp.86, the formation of a Co-C bond linking the bishomocubane cage to the corrinoid could be inferred. It is likely that a Co(I) oxidation state would be needed to enable the chlordecone attack (Schrauzer and Katz, 1978). In 1978, Schrauzer and Katz apparently detected [Co]-C_3_Cl_3_H_2_ fragments within the abiotic reaction mixture of chlordecone with vitamin B_12_, supporting this assumption.

In addition, it is known that several chemical transformations of chlordecone involving vitamin B_12_ reduced under a Co(I) oxidation state by strong reducing agents generate the same TP profile (Schrauzer and Katz, 1978; Ranguin et al., 2017; Chevallier et al., 2018, 2019). In our hands, use of chloro(pyridine)cobaloxime, known to be a good model of vitamin B_12_ (Schrauzer and Katz, 1978; Terán et al., 2018; Pizarro et al., 2019) also led to the same diversity of chlordecone TPs in presence of a strong reducing agent (Figure S6). It supports the hypothesis that several cobalt complexes containing a corrin ring could afford the same chlordecone TPs, once reduced.

The present results show that microbial degradation of chlordecone mediated by *Citrobacter* sp.86 is clearly correlated to the production of cobalamin derivatives. Chemical degradation using vitamin B_12_ or cobaloxime in aqueous solution also leads to the same TPs (Schrauzer and Katz, 1978; Jablonski et al., 1996; Ranguin et al., 2017; Chevallier et al., 2018, 2019). Taken together, these observations suggest that microbial and chemical degradations share strong similarities in their mechanistic pathway. These conclusions are apparently not supported by some of our previous findings obtained from carbon specific isotope analysis (CSIA). Indeed, the carbon isotopic enrichment factors observed for microbiological and vitamin B_12_-mediated degradations differed significantly, suggesting two distinct mechanisms (Chevallier et al., 2018). However, the strong pH variation (pH 7 for microbiological cultures and pH 12 for chemical conditions) could also explain the difference in the ^13^C isotopic signatures. For instance, Heckel and co-workers observed that a change from acidic to basic conditions led to a switch in dechlorination mechanisms during the chemical degradation of trichloroethene mediated by vitamin B_12_ reduced under a Co(I) state (Heckel et al., 2018).

Furthermore, it is possible that biologically produced corrinoids could also be responsible for chlordecone degradation performed by anaerobic bacteria and archaea described in other studies. Thereby, the chlordecone degradation by the methanogen *M. thermophila* seemed to be mediated by corrinoids (Jablonski et al., 1996). Interestingly, consortia 86 and 92 able to degrade chlordecone contained, among others, *Desulfovibrio*, *Pleomorphomonas*, and *Sporomusa* species, possessing cobalamin biosynthesis genes (Chaussonnerie et al., 2016). Among these bacteria, *Desulfovibrio* sp.86 was isolated and showed to degrade chlordecone along with the same TPs profile as observed in presence of *Citrobacter* sp.86 (Della-Negra et al., 2020). In the same way, in microcosms amended with chlordecone, no known obligate organohalide respiring bacteria were observed, whereas enrichment with *Desulfovibrio*, *Sporomusa*, and *Geobacter* species or methanogens were noticed (Lomheim et al., 2020). Still, in this case, these bacteria and archaea could be corrinoid-producers.

### Lindane Transformation by *Citrobacter* sp.86 Wild-Type and Mutant Strains

Lindane (γ-hexachlorocyclohexane) dechlorination by *C. freundii* under anaerobic conditions has been already described (Jagnow et al., 1977). Remarkably, as it is the case for the degradation of chlordecone by *Citrobacter* sp.86, it was noticed that dechlorination of lindane may not be related to *C. freundii* growth. In this context, we tested whether *Citrobacter* sp.86, which is a different species than *C. freundii*, was intrinsically able to dechlorinate lindane and if this dechlorination was mediated by cobalamin or corrinoids.

**Figure 4:**
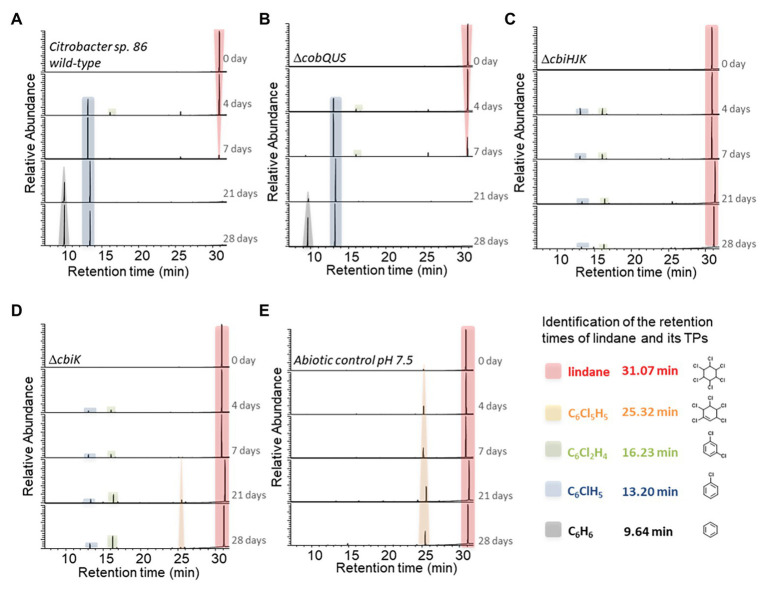
Transformation of lindane in *Citrobacter* sp.86 wild-type and mutant strain cultures over the time. HS-GC-MS extracted ion chromatograms (m/z = 181, 147, 146, 112, and 78, searched for each condition) of sampled cultures at selected times incubated with **(A)** *Citrobacter* sp.86 wild-type, **(B)** Δ*cobQUS* mutant strain, **(C)** Δ*cbiHJK* mutant strain, **(D)** Δ*cbiK* mutant strain, and **(E)** without bacteria (abiotic control). Numeric data of peak areas are available in Supplementary Material. The various mutant strains and the negative control (abiotic control) were incubated under the same conditions. For more clarity, a single chromatogram is displayed among the duplicates at each selected time.

Under the same incubation conditions as for chlordecone, in another set of experiments, lindane did not impede the growth of *Citrobacter* sp.86 (Figure S5). Lindane degradation was observed with the concomitant formation of benzene and chlorobenzene as major TPs, (Figure 4). Trace level of γ-tetrachlorocyclohexene was also detected during lindane degradation operated by *Citrobacter* sp.86 (Supplementary Material). After 28 days, no more lindane in the culture was detected by HS-GC-MS. In contrast, after 42-day incubation, chlordecone was still detected by GC-MS analysis (Figure 3). The higher solubility in water of lindane (7–9 mg/L at 25°C and pH 7, Saley et al., 1982) compared to chlordecone (1–2 mg/L at 25°C and pH 7, Dawson et al., 1979) may account for its higher degradability (at the same molar ratio) by the *Citrobacter* sp.86 cultures.

Chlorobenzene, benzene, and/or γ-tetrachlorocyclohexene have already been described as TPs observed in anaerobic degradation of lindane by *C. freundii*, sulfate-reducing bacteria including *D. gigas*, *D. multivorans*, and also in bacterial consortia enriched in *Pelobacter* (Jagnow et al., 1977; Boyle et al., 1999; Badea et al., 2009; Qiao et al., 2020). It is likely that these TPs appeared by a two-step process involving two sequential dichloroelimination reactions, followed by another dichloroelimination to produce benzene or a hydrodechlorination to generate chlorobenzene (Zhang et al., 2014; Qiao et al., 2020; Figure S1B). After *C. freundii* (Jagnow et al., 1977), *Citrobacter* sp.86 is the second species of this genus reported to degrade lindane, and this degradation most likely occurs in the same way as in other anaerobic bacteria. These bacteria dechlorinate lindane cometabolically, with for instance glucose, lactate, or pyruvate as suitable carbon sources.

We also observed that *Citrobacter* sp.86 Δ*cobQUS* degraded lindane just like the wild-type (Figure 4B). In contrast, *Citrobacter* sp.86 Δ*cbiHJK* and *Citrobacter* sp.86 Δ*cbiK* were unable to quantitatively dechlorinate lindane (Figures 4C,D). Low levels of chlorobenzene and 1,3-dichlorobenzene as well as trace amount of 1,3,4,5,6-pentachlorocyclohex-1-ene were observed, but GC-MS peak areas were insignificant compared to the *Citrobacter* sp.86 wild-type strain and Δ*cobQUS* mutant strain. In comparison, abiotic controls showed a significant formation of 1,3,4,5,6-pentachlorocyclohex-1-ene while lindane remained definitely predominant after 28 days. This TP has been previously reported during the photolysis of lindane (Zaleska et al., 2000). As all degradation experiments were performed in daylight, we assumed that an additional slow photodegradation process was taking place. The rate of photodegradation would depend on the prevalence of other competing degradation pathways.

**Figure 5:**
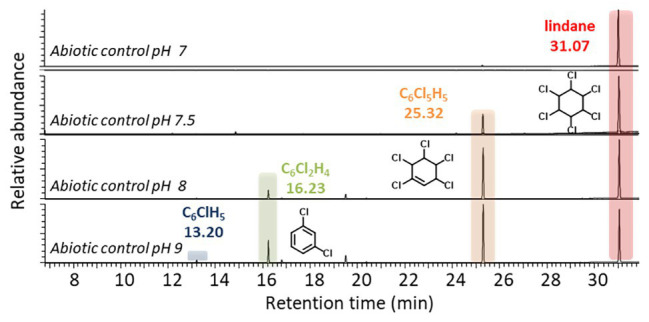
Abiotic transformation of lindane in neutral and basic conditions. HS-GC-MS extracted ion chromatograms (m/z = 181, 147, 146, 112, and 78, searched for each condition) after 21 days of abiotic cultures at different pH: 7, 7.5, 8, and 9. Numeric data of peak areas are available in Supplementary Material.

An additional abiotic pH-dependence study of lindane dechlorination showed that 1,3,4,5,6-pentachlorocyclohex-1-ene, 1,3-dichlorobenzene, and chlorobenzenewere spontaneously produced, albeit at low rate, when the pH of the incubation medium was set at more basic values (pH ≥ 8), as it has already been described (Figure 5; Hiskia et al., 1997). The possible photodegradadation process and the pH-dependent degradation phenomenon could also explain the detection of 1,3-dichlorobenzene and chlorobenzene in mutants *Citrobacter* sp.86 Δ*cbiHJK* and *Citrobacter* sp.86 Δ*cbiK*.

These results show that corrinoids synthesized by *Citrobacter* sp.86 as well as *Citrobacter* sp.86 Δ*cobQUS* are involved in the most prominent lindane degradation pathway. As for chlordecone, a complete cobalamin molecule was not needed for lindane degradation. Also, by abiotic processes, Marks et al. (1989) showed high activity of lindane dechlorination by cobinamides reduced with dithiothreitol. In their work, the authors tested a variety of porphyrins and corrins for catalysis of lindane dehalogenation. They showed the importance of the cobalt ion in the tetrapyrrole ring. Also, dehalogenation activity of cobinamides was about 8-fold higher than that of cobalamin. These authors suggested that the lower ligand could sterically hinder the approach of lindane to the cobalt ion at the center of the corrin ring. It could also be that the absence of the lower base coordination results in an increase of the oxido-reduction potential of cobinamide compared to cobalamin, which becomes easier to reduce (Dereven’kov et al., 2016).

Finally, we postulate that the implication of corrinoids, synthesized by *Citrobacter* sp.86, in lindane dechlorination as shown in this study could be extended not only to *C. freundii* but also to other bacteria that anaerobically degrade lindane such as *D. gigas*, *D. multivorans*, and *Clostridium* (Macrae et al., 1969; Jagnow et al., 1977; Ohisa and Yamaguchi, 1978; Boyle et al., 1999; Badea et al., 2009; Mehboob et al., 2013). In the same way, corrinoids could be involved in lindane degradation by consortia with the enrichment of *Pelobacter* within the dehalogenation process (Qiao et al., 2020). All these bacteria (i) are fermenters, (ii) transform lindane co-metabolically, and (iii) seem to be able to produce cobalamin (Roth et al., 1996; Shelton et al., 2019).

### Targeting Microbial Corrinoids as Specific Tools for Organochlorine Pesticides Degradation

Under abiotic conditions, involvement of corrinoids in transformation and dechlorination of compounds including chlordecone and lindane has been well-documented (Schrauzer and Katz, 1978; Marks et al., 1989; Jablonski et al., 1996; Rodríguez-Garrido et al., 2004; Ranguin et al., 2017; Chevallier et al., 2018, 2019; Guo and Chen, 2018; Pizarro et al., 2019). Under biotic conditions, involvement of corrinoids as cofactors in reductive dehalogenases found in organohalide respiring bacteria that transform a variety of organochlorides pollutants was also known (Schubert et al., 2018). Moreover, a dechlorination process was observed in non-organohalide-respiring bacteria and under these conditions, the implication of protein-free corrinoids was already suggested (Jagnow et al., 1977; Badea et al., 2009; Lomheim et al., 2020). This last point was confirmed in that study.

From an environmental point of view, it is highly plausible that corrinoids produced by bacteria could have a significant impact on the natural biodegradation of organochlorine compounds under anaerobic conditions. Pools of cobalamin are produced *de novo* and subject to complex trade-offs for salvage and remodeling in terrestrial and marine ecosystems (Heal et al., 2017; Lu et al., 2020). In contaminated sites, biostimulation of bacteria producing corrinoids could be a method of depollution (Guo and Chen, 2018). Although, the harsh reductive conditions, which are required for corrinoid-mediated chlordecone and lindane biotransformations in the laboratory may not easily be met in polluted agricultural soils, the presence of degradation products in natural environments (Chevallier et al., 2019) leaves this possibility open.

On a smaller and easier to control scale, for example in reactors, biodegradation could be performed using bacteria producing corrinoids at high yield. Though anaerobic bacteria, for example from the genus *Desulfovibrio*, are known as producers of corrinoids (Men et al., 2014, 2015), bacteria from the genus *Citrobacter* like for instance *Citrobacter* sp.86 could be of great interest in co-metabolic degradations. As we show here, this facultative anaerobic bacterium that displays a versatile anaerobic metabolism involving cobalamin (e.g., synthesis of building blocks, degradation of glycerol, propanediol, ethanolamine, and glutamate…) can be easily manipulated genetically. Tools of the synthetic biology toolbox (Dvořák et al., 2017) like knockin and knockout strategies could therefore be implemented in *Citrobacter* sp.86 to determine the optimal corrinoids, enhance their production and test for further improvements in lindane, chlordecone, or other organochlorine transformations in the perspective of possible bioremediation improvement.

## Conclusion

In this study, we show clearly, through the examination of *Citrobacter* sp.86 knockout mutant strains defective in cobalamin biosynthesis, that corrinoids are involved in chlordecone and lindane biotransfomations. This provides new information on the mechanistic issues of chlordecone transformation. In addition, the present work highlights the importance of cobalt insertion into the tetrapyrrole ring for these transformations.

Biotransformations mediated by corrinoids produced by *Citrobacter* sp.86 are not substrate-specific processes as shown here using different organochlorines compounds such as chlordecone (bishomocubane structure) and lindane (γ-hexachlorocyclohexane). Further investigations could extend the present degradation spectrum to other organochlorines. Furthermore, it would also be interesting to know how this bacterium is able to reduce its corrinoids to Co(I), an oxidation state that might be required for the ring-opening dechlorination of chlordecone.

## Data Availability Statement

The original contributions presented in the study are included in the article/Supplementary Material, further inquiries can be directed to the corresponding authors.

## Author Contributions

DP and NF conceived the study, designed the experiments, and supervised the microbiology and molecular biology part. AB, OD-N, SC, VD, DM, EU, and NF performed the experiments. OD-N, DM, P-LS, and NF carried out the data analysis. OD-N, CF, DP, and NF wrote the paper. AB, OD-N, and SC assisted in the formatting of the Figures. SC, P-LS, JW, and DP helped to revise the manuscript. P-LS supervised and developed the analytical and monitoring methods. All authors participated in the discussion of the manuscript, and agreed on the final content.

### Conflict of Interest

The authors declare that the research was conducted in the absence of any commercial or financial relationships that could be construed as a potential conflict of interest.

## References

[ref1] Álvarez A. Yañez M. L. Benimeli C. S. Amoroso M. J. (2012). Maize plants (*Zea mays*) root exudates enhance lindane removal by native *Streptomyces* strains. Int. Biodeterior. Biodegradation 66, 14–18. 10.1016/j.ibiod.2011.10.001

[ref2] Badea S. -L. Vogt C. Weber S. Danet A. -F. Richnow H. -H. (2009). Stable isotope fractionation of γ-hexachlorocyclohexane (Lindane) during reductive dechlorination by two strains of sulfate-reducing bacteria. Environ. Sci. Technol. 43, 3155–3161. 10.1021/es801284m, PMID: 19534128

[ref3] Bashir S. Kuntze K. Vogt C. Nijenhuis I. (2018). Anaerobic biotransformation of hexachlorocyclohexane isomers by *Dehalococcoides* species and an enrichment culture. Biodegradation 29, 409–418. 10.1007/s10532-018-9838-9, PMID: 29916096

[ref4] Belghit H. Colas C. Bristeau S. Mouvet C. Maunit B. (2015). Liquid chromatography-high-resolution mass spectrometry for identifying aqueous chlordecone hydrate dechlorinated transformation products formed by reaction with zero-valent iron. Int. J. Environ. Anal. Chem. 95, 93–105. 10.1080/03067319.2014.994615

[ref5] Böltner D. Morillas S. M. Ramos J. -L. (2005). 16S rDNA phylogeny and distribution of Lin genes in novel hexachlorocyclohexane-degrading *Sphingomonas* strains. Environ. Microbiol. 7, 1329–1338. 10.1111/j.1462-5822.2005.00820.x, PMID: 16104856

[ref7] Boyle A. W. Phelps C. D. Young L. Y. (1999). Isolation from estuarine sediments of a desulfovibrio strain which can grow on lactate coupled to the reductive dehalogenation of 2,4,6-Tribromophenol. Appl. Environ. Microbiol. 65, 1133–1340. 10.1128/AEM.65.3.1133-1140.1999, PMID: 10049873 PMC91154

[ref8] Cabidoche Y. -M. Achard R. Cattan P. Clermont-Dauphin C. Massat F. Sansoulet J. (2009). Long-term pollution by Chlordecone of tropical volcanic soils in the French West Indies: a simple leaching model accounts for current residue. Environ. Pollut. 157, 1697–1705. 10.1016/j.envpol.2008.12.015, PMID: 19167793

[ref9] Camacho-Pérez B. Ríos-Leal E. Rinderknecht-Seijas N. Poggi-Varaldo H. M. (2012). Enzymes involved in the biodegradation of hexachlorocyclohexane: a mini review. J. Environ. Manag. 95, 306–318. 10.1016/j.jenvman.2011.06.047, PMID: 21992990

[ref10] Chaussonnerie S. Saaidi P. -L. Ugarte E. Barbance A. Fossey A. Barbe V.. (2016). Microbial degradation of a recalcitrant pesticide: chlordecone. Front. Microbiol. 7:2025. 10.3389/fmicb.2016.02025, PMID: 28066351 PMC5167691

[ref11] Chevallier M. L. Cooper M. Kümmel S. Barbance A. Le Paslier D. Richnow H. H.. (2018). Distinct carbon isotope fractionation signatures during biotic and abiotic reductive transformation of chlordecone. Environ. Sci. Technol. 52, 3615–3624. 10.1021/acs.est.7b05394, PMID: 29473745

[ref12] Chevallier M. L. Della-Negra O. Chaussonnerie S. Barbance A. Muselet D. Lagarde F.. (2019). Natural chlordecone degradation revealed by numerous transformation products characterized in key French West Indies environmental compartments. Environ. Sci. Technol. 53, 6133–6143. 10.1021/acs.est.8b06305, PMID: 31082212

[ref13] Cuozzo S. A. Sineli P. E. Davila Costa J. Tortella G. (2017). *Streptomyces* sp. is a powerful biotechnological tool for the biodegradation of HCH isomers: biochemical and molecular basis. Crit. Rev. Biotechnol. 38, 719–728. 10.1080/07388551.2017.1398133, PMID: 29124958

[ref14] Datsenko K. A. Wanner B. L. (2000). One-step inactivation of chromosomal genes in *Escherichia coli* K-12 using PCR products. Proc. Natl. Acad. Sci. 97, 6640–6645. 10.1073/pnas.120163297, PMID: 10829079 PMC18686

[ref15] Dawson G. W. Weimer W. C. Shupe S. J. (1979). Kepone - A case study of a persistent material. The American Institute of Chemical Engineers (AIChE) Symposium Series. 75, 366–374.

[ref16] Della-Negra O. Chaussonnerie S. Fonknechten N. Barbance A. Muselet D. Martin D. E.. (2020). Transformation of the recalcitrant pesticide chlordecone by *Desulfovibrio* sp.86 with a switch from ring-opening dechlorination to reductive sulfidation activity. Sci. Rep. 10:13545. 10.1038/s41598-020-70124-9, PMID: 32782344 PMC7419502

[ref17] Dereumeaux C. Saoudi A. Guldner L. Pecheux M. Chesneau J. Thomé J. -P. (2019). Chlordecone and organochlorine compound levels in the French West Indies population in 2013–2014. Environ. Sci. Pollut. Res. Int.. 10.1007/s11356-019-07181-9 [Epub ahead of print]. 31884530

[ref18] Dereven’kov I. A. Salnikov D. S. Silaghi-Dumitrescu R. Makarov S. V. Koifman O. I. (2016). Redox chemistry of cobalamin and its derivatives. Coord. Chem. Rev. 309, 68–83. 10.1016/j.ccr.2015.11.001

[ref19] Dogra C. Raina V. Pal R. Suar M. Lal S. Gartemann K. -H.. (2004). Organization of lin genes and IS6100 among different strains of hexachlorocyclohexane-degrading *Sphingomonas Paucimobilis*: evidence for horizontal gene transfer. J. Bacteriol. 186, 2225–2235. 10.1128/JB.186.8.2225-2235.2004, PMID: 15060023 PMC412113

[ref20] Doublet B. Douard G. Targant H. Meunier D. Madec J. -Y. Cloeckaert A. (2008). Antibiotic marker modifications of λ red and FLP helper plasmids, PKD46 and PCP20, for inactivation of chromosomal genes using PCR products in multidrug-resistant strains. J. Microbiol. Methods 75, 359–361. 10.1016/j.mimet.2008.06.010, PMID: 18619499

[ref21] Dvořák P. Nikel P. I. Damborský J. de Lorenzo V. (2017). Bioremediation 3.0: engineering pollutant-removing bacteria in the times of systemic biology. Biotechnol. Adv. 35, 845–866. 10.1016/j.biotechadv.2017.08.001, PMID: 28789939

[ref22] Elango V. Kurtz H. D. Anderson C. Freedman D. L. (2011). Use of γ-hexachlorocyclohexane as a terminal electron acceptor by an anaerobic enrichment culture. J. Hazard. Mater. 197, 204–210. 10.1016/j.jhazmat.2011.09.080, PMID: 21983168

[ref23] Endo R. Kamakura M. Miyauchi K. Fukuda M. Ohtsubo Y. Tsuda M.. (2005). Identification and characterization of genes involved in the downstream degradation pathway of *γ*-hexachlorocyclohexane in *Sphingomonas paucimobilis* UT26. J. Bacteriol. 187, 847–853. 10.1128/JB.187.3.847-853.2005, PMID: 15659662 PMC545726

[ref24] Epstein S. S. (1978). Kepone-Hazard Evaluation. Sci. Total Environ. 9, 1–62. 10.1016/0048-9697(78)90002-5, PMID: 74851

[ref25] Fernández-Bayo F. D. Saison C. Voltz M. Disko U. Hofmann D. Berns A. E. (2013). Chlordecone fate and mineralisation in a tropical soil (andosol) microcosm under aerobic conditions. Sci. Total Environ. 463-434, 395–203. 10.1016/j.scitotenv.2013.06.044, PMID: 23827360

[ref26] Fincker M. Spormann A. M. (2017). Biochemistry of catabolic reductive dehalogenation. Annu. Rev. Biochem. 86, 357–386. 10.1146/annurev-biochem-061516-044829, PMID: 28654328

[ref27] Fonseca M. V. Escalante-Semerena J. C. (2000). Reduction of cob(III)alamin to cob(II)alamin in *Salmonella enterica* Serovar Typhimurium LT2. J. Bacteriol. 182, 4304–4309. 10.1128/JB.182.15.4304-4309.2000, PMID: 10894741 PMC101946

[ref28] George S. E. Claxton L. -D. (1988). Biotransformation of chlordecone by *Pseudomonas* species. Xenobiotica 18, 407–416. 10.3109/00498258809041677, PMID: 2456645

[ref29] Guo M. Chen Y. (2018). Coenzyme cobalamin: biosynthesis, overproduction and its application in dehalogenation-a review. Rev. Environ. Sci. Biotechnol. 17, 259–284. 10.1007/s11157-018-9461-6

[ref30] Heal K. R. Qin W. Ribalet F. Bertagnolli A. D. Coyote-Maestas W. Hmelo L. R.. (2017). Two distinct pools of B12 analogs reveal community interdependencies in the ocean. Proc. Natl. Acad. Sci. U. S. A. 114, 364–369. 10.1073/pnas.1608462114, PMID: 28028206 PMC5240700

[ref31] Heckel B. McNeill K. Elsner M. (2018). Chlorinated ethene reactivity with vitamin B12 is governed by cobalamin chloroethylcarbanions as crossroads of competing pathways. ACS Catal. 8, 3045–3066. 10.1021/acscatal.7b02945

[ref32] Hiskia A. Mylonas A. Tsipi D. Papaconstantinou E. (1997). Photocatalytic degradation of Lindane in aqueous solution. Pestic. Sci. 50, 171–174. 10.1002/(SICI)1096-9063(199706)50:2<171::AID-PS565>3.0.CO;2-H

[ref33] Huggett R. J. Bender M. E. (1980). Kepone in the James River. Environ. Sci. Technol. 14, 918–923. 10.1021/es60168a001, PMID: 22296535

[ref34] Jablonski P. E. Pheasant D. H. Ferry J. G. (1996). Conversion of Kepone by *Methanosarcina Thermophila*. FEMS Microbiol. Lett. 139, 169–173. 10.1111/j.1574-6968.1996.tb08198.x

[ref35] Jagnow G. Haider K. Ellwardt P. C. (1977). Anaerobic dechlorination and degradation of hexachlorocyclohexane isomers by anaerobic and facultative anaerobic bacteria. Arch. Microbiol. 115, 285–292. 10.1007/BF00446454, PMID: 74989

[ref36] Jennings A. A. Li Z. (2015). Residential surface soil guidance applied worldwide to the pesticides added to the Stockholm convention in 2009 and 2011. J. Environ. Manag. 160, 226–240. 10.1016/j.jenvman.2015.06.020, PMID: 26144561

[ref37] Jeter R. M. Olivera B. M. Roth J. R. (1984). Salmonella Typhimurium synthesizes cobalamin (vitamin B12) de novo under anaerobic growth conditions. J. Bacteriol. 159, 206–213. 10.1128/JB.159.1.206-213.1984, PMID: 6376471 PMC215614

[ref38] Kumari R. Subudhi S. Suar M. Dhingra G. Raina V. Dogra C.. (2002). Cloning and characterization of Lin genes responsible for the degradation of hexachlorocyclohexane isomers by *Sphingomonas Paucimobilis* strain B90. Appl. Environ. Microbiol. 68, 6021–6028. 10.1128/AEM.68.12.6021-6028.2002, PMID: 12450824 PMC134425

[ref39] Lal R. Dogra C. Malhotra S. Sharma P. Pal R. (2006). Diversity, distribution and divergence of Lin genes in hexachlorocyclohexane-degrading *Sphingomonads*. Trends Biotechnol. 24, 121–130. 10.1016/j.tibtech.2006.01.005, PMID: 16473421

[ref40] Lal R. Pandey G. Sharma P. Kumari K. Malhotra S. Pandey R.. (2010). Biochemistry of microbial degradation of hexachlorocyclohexane and prospects for bioremediation. Microbiol. Mol. Biol. Rev. 74, 58–80. 10.1128/MMBR.00029-09, PMID: 20197499 PMC2832351

[ref41] Laquitaine L. Durimel A. de Alencastro L. F. Jean-Marius C. Gros O. Gaspard S. (2016). Biodegradability of HCH in agricultural soils from Guadeloupe (French West Indies): identification of the Lin genes involved in the HCH degradation pathway. Environ. Sci. Pollut. Res. 23, 120–127. 10.1007/s11356-015-5875-7, PMID: 26686518

[ref42] Le Déaut J-Y. Procaccia C. (2009). Impacts de l’utilisation de la chlordécone et des pesticides aux Antilles: bilan et perspectives d’évolution. Office parlementaire d’évaluation des choix scientifiques et technologiques Rapport n° 487 (2008–2009). Availavle at: https://www.senat.fr/rap/r08-487/r08-487.html

[ref43] Lesueur-Jannoyer M. Cattan P. Woignier T. Clostre F. (2016). Crisis management of chronic pollution: Contaminated soil and human health. Boca Raton, US: CRC Press.

[ref44] Lomheim L. Laquitaine L. Rambinaising S. Flick R. Starostine A. Jean-Marius C.. (2020). Evidence for extensive anaerobic dechlorination and transformation of the pesticide chlordecone (C10Cl10O) by indigenous microbes in microcosms from Guadeloupe soil. PLoS One 15:e0231219. 10.1371/journal.pone.0231219, PMID: 32282845 PMC7153859

[ref45] Lu X. Heal K. R. Ingalls A. E. Doxey A. C. Neufeld J. D. (2020). Metagenomic and chemical characterization of soil Cobalamin production. ISME J. 14, 53–66. 10.1038/s41396-019-0502-0, PMID: 31492962 PMC6908642

[ref46] Macrae I. C. Raghu K. Bautista E. M. (1969). Anaerobic degradation of the insecticide lindane by *Clostridium* Sp. Nature 221, 859–860. 10.1038/221859a0, PMID: 4179478

[ref47] Marks T. S. Allpress J. D. Maule A. (1989). Dehalogenation of lindane by a variety of porphyrins and corrins. Appl. Environ. Microbiol. 55, 1258–1261. 10.1128/AEM.55.5.1258-1261.1989, PMID: 2474266 PMC184286

[ref48] Maudouit M. Rochoy M. (2019). Systematic review of the impact of chlordecone on human health in the French West Indies. Therapie 74, 611–625. 10.1016/j.therap.2019.01.010, PMID: 31088689

[ref49] Mehboob F. Langenhoff A. A. M. Schraa G. Stams A. J. M. (2013). “Anaerobic degradation of lindane and other HCH isomers” in Management of microbial resources in the environment. eds. Malik A. Grohmann E. Alves M. (Dordrecht: Springer), 495–521.

[ref50] Men Y. Seth E. C. Yi S. Allen R. H. Taga M. E. Alvarez-Cohen L. (2014). Sustainable growth of *Dehalococcoides mccartyi* 195 by corrinoid salvaging and remodeling in defined lactate-fermenting consortia. Appl. Environ. Microbiol. 80, 2133–2141. 10.1128/AEM.03477-13, PMID: 24463969 PMC3993147

[ref51] Men Y. Seth E. C. Yi S. Crofts T. S. Allen R. H. Taga M. E.. (2015). Identification of specific corrinoids reveals corrinoid modification in dechlorinating microbial communities. Environ. Microbiol. 17, 4873–4884. 10.1111/1462-2920.12500, PMID: 24803319 PMC4942503

[ref52] Middeldorp P. J. M. Jaspers M. Zehnder A. J. B. Schraa G. (1996). Biotransformation of α-, β-, γ-, and δ-hexachlorocyclohexane under methanogenic conditions. Environ. Sci. Technol. 30, 2345–2349. 10.1021/es950782+

[ref53] Multigner L. Rouget F. Costet N. Monfort C. Blanchet P. Kadhel P. (2018). Chlordécone: un perturbateur endocrinien emblématique affectant les Antilles Françaises. Bull Epidemiol. Hebd. 22-23, 480–485. http://invs.santepubliquefrance.fr/beh/2018/22-23/2018_22-23_4.html (Accessed October 27, 2020).

[ref54] Nagata Y. Endo R. Ito M. Ohtsubo Y. Tsuda M. (2007). Aerobic degradation of lindane (γ-hexachlorocyclohexane) in bacteria and its biochemical and molecular basis. Appl. Microbiol. Biotechnol. 76, 741–752. 10.1007/s00253-007-1066-x, PMID: 17634937

[ref55] Nichols M. M. (1990). Sedimentologic fate and cycling of kepone in an estuarine system: example from the James River estuary. Sci. Total Environ. 97-98, 407–440. 10.1016/0048-9697(90)90254-R

[ref56] Nichols M. M. Cutshall N. H. (1981). Tracing kepone contamination in James estuary sediments. Re’un. Cons. Int. Explor. Mer. 137.

[ref57] Nolan K. Kamrath J. Levitt J. (2012). Lindane toxicity: a comprehensive review of the medical literature. Pediatr. Dermatol. 29, 141–146. 10.1111/j.1525-1470.2011.01519.x, PMID: 21995612

[ref58] Ohisa N. Yamaguchi M. (1978). Degradation of gamma-BHC in flooded soils enriched with peptone. Agric. Biol. Chem. 42, 1983–1987. 10.1080/00021369.1978.10863297

[ref59] Orndorff S. A. Colwell R. R. (1980). Microbial transformation of kepone. Appl. Environ. Microbiol. 39, 398–406. 10.1128/AEM.39.2.398-406.1980, PMID: 6155103 PMC291344

[ref61] Pizarro S. Gallardo M. Gajardo F. Delgadillo A. (2019). Electrochemical reduction of lindane using a cobaloxime containing electron-withdrawing groups. Inorg. Chem. Commun. 99, 164–166. 10.1016/j.inoche.2018.10.014

[ref62] Qiao W. Jácome L. A. P. Tang X. Lomheim L. Yang M. I. Gaspard S.. (2020). Microbial communities associated with sustained anaerobic reductive dechlorination of α-, β-, γ-, and δ-hexachlorocyclohexane isomers to monochlorobenzene and benzene. Environ. Sci. Technol. 54, 255–265. 10.1021/acs.est.9b05558, PMID: 31830788

[ref63] Quintero J. C. Moreira M. T. Feijoo G. Lema J. M. (2005). Anaerobic degradation of hexachlorocyclohexane isomers in liquid and soil slurry systems. Chemosphere 61, 528–536. 10.1016/j.chemosphere.2005.02.010, PMID: 16202806

[ref64] Ranguin R. Durimel A. Karioua R. Gaspard S. (2017). Study of chlordecone desorption from activated carbons and subsequent dechlorination by reduced cobalamin. Environ. Sci. Pollut. Res. 24, 25488–25499. 10.1007/s11356-017-9542-z, PMID: 28699005

[ref66] Robles-González I. V. Fava F. Poggi-Varaldo H. M. (2008). A review on slurry bioreactors for bioremediation of soils and sediments. Microb. Cell Factories 7, 1–16. 10.1186/1475-2859-7-5, PMID: 18312630 PMC2292675

[ref67] Rodríguez-Garrido B. Camps Arbestain M. Macías F. (2004). Reductive dechlorination of α-, β-, δ-, and γ-hexachlorocyclohexane isomers by hydroxocobalamin in the presence of either dithiothreitol or titanium(III) citrate as reducing agents. Environ. Sci. Technol. 38, 5046–5052. 10.1021/es030153x, PMID: 15506197

[ref68] Roth J. R. Lawrence J. G. Bobik T. A. (1996). Cobalamin (coenzyme B12): synthesis and biological significance. Annu. Rev. Microbiol. 50, 137–181. 10.1146/annurev.micro.50.1.137, PMID: 8905078

[ref69] Saez J. M. Alvarez A. Fuentes M. S. Amoroso M. J. Benimeli C. S. (2017). “An overview on microbial degradation of Lindane” in *microbe-induced degradation of pesticides* ed. Singh S. N. (Springer, Cham).

[ref70] Sakakibara F. Takagi K. Kataoka R. Kiyota H. Sato Y. Okada S. (2011). Isolation and identification of dieldrin-degrading *Pseudonocardia* sp. strain KSF27 using a soil–charcoal perfusion method with aldrin trans-diol as a structural analog of dieldrin. Biochem. Biophys. Res. Commun. 411, 76–81. 10.1016/j.bbrc.2011.06.096, PMID: 21708128

[ref71] Saley F. Y. Dickson L. Rodgers J. H. Jr. (1982). Fate of lindane in the aquatic environment: rate constants of physical and chemical processes. Environ. Toxicol. Chem. 1, 289–297. 10.1002/etc.5620010404

[ref73] Schrauzer G. N. Katz R. N. (1978). Reductive dechlorination and degradation of mirex and kepone with vitamin B12s. Bioinorg. Chem. 9, 123–142. 10.1016/S0006-3061(00)80285-9, PMID: 81074

[ref74] Schubert T. Adrian L. Sawers R. G. Diekert G. (2018). Organohalide respiratory chains: composition, topology and key enzymes. FEMS Microbiol. Ecol. 1:94. 10.1093/femsec/fiy035, PMID: 29718172

[ref75] Senoo K. Wada H. (1989). Isolation and identification of an aerobic γ-HCH-decomposing bacterium from soil. J. Soil Sci. Plant Nutr. 35, 79–87. 10.1080/00380768.1989.10434739

[ref76] Shelton A. N. Seth E. C. Mok K. C. Han A. W. Jackson S. N. Haft D. R.. (2019). Uneven distribution of cobamide biosynthesis and dependence in bacteria predicted by comparative genomics. ISME J. 13, 789–804. 10.1038/s41396-018-0304-9, PMID: 30429574 PMC6461909

[ref77] Terán J. E. Zambrano C. H. Mora J. R. Rincón L. Torres F. J. (2018). Theoretical investigation of the mechanism for the reductive dehalogenation of methyl halides mediated by the CoI-based compounds cobalamin and cobaloxime. J. Mol. Model. 24:316. 10.1007/s00894-018-3844-z, PMID: 30338391

[ref78] Trotman R. C. Nichols M. M. (1978). *Kepone in bed sediments of the James River Estuary*. Special scientific report No. 91. Virginia Institute of Marine Science: College of William and Mary.

[ref79] Unger M. Vadas G. (2017). *Kepone in the James River Estuary: past, current and future trends*. Virginia Institute of Marine Science: William & Mary.

[ref80] van der Ploeg J. van Hall G. Janssen D. B. (1991). Characterization of the haloacid dehalogenase from xanthobacter autotrophicus GJ10 and sequencing of the DhlB gene. J. Bacteriol. 173, 7925–7933. 10.1128/jb.173.24.7925-7933.1991, PMID: 1744048 PMC212586

[ref81] Vijgen J. de Borst B. Weber R. Stobiecki T. Forter M. (2019). HCH and lindane contaminated sites: European and global need for a permanent solution for a long-time neglected issue. Environ. Pollut. 248, 696–705. 10.1016/j.envpol.2019.02.029, PMID: 30849587

[ref82] Vilardebo A. Beugnon M. Melin P. Lecoq J. Aubert B. (1974). Chlordecone et autres insecticides dans la lutte contre le charançon du bananier *Cosmopolites sordidus* Germ. Fruits 29, 267–278.

[ref83] Wacławek S. Silvestri D. Hrabák P. Padil V. V. T. Torres-Mendieta R. Wacławek M.. (2019). Chemical oxidation and reduction of hexachlorocyclohexanes: a review. Water Res. 162, 302–319. 10.1016/j.watres.2019.06.072, PMID: 31288141

[ref84] Wilson N. K. Zehr R. D. (1978). Structures of some Kepone photoproducts and related chlorinated pentacyclodecanes by carbon-13 and proton nuclear magnetic resonance. J. Organomet. Chem. 44, 1278–1282. 10.1021/jo01322a020

[ref85] Zaleska A. Hupka J. Wiergowski M. Biziuk M. (2000). Photocatalytic degradation of lindane, p,p'-DDT and methoxychlor in an aqueous environment. J. Photochem. Photobiol. 135, 213–220. 10.1016/S1010-6030(00)00296-3

[ref86] Zhang N. Bashir S. Qin J. Schindelka J. Fischer A. Nijenhuis I.. (2014). Compound specific stable isotope analysis (CSIA) to characterize transformation mechanisms of α-hexachlorocyclohexane. J. Hazard. Mater. 280, 750–757. 10.1016/j.jhazmat.2014.08.046, PMID: 25238192

[ref87] Zhang W. Lin Z. Pang S. Bhatt P. Chen S. (2020). Insights into the biodegradation of lindane (γ-hexachlorocyclohexane) using a microbial system. Front. Microbiol. 11:522. 10.3389/fmicb.2020.00522, PMID: 32292398 PMC7119470

