# Supplementary material for: Genetic Analysis of Citrobacter sp.86 Reveals Involvement of Corrinoids in Chlordecone and Lindane Biotransformations
Source: Front Microbiol. 2020 Nov 9;11:590061. doi: 10.3389/fmicb.2020.590061 (PMC7680753; doi:10.3389/fmicb.2020.590061)
Supplement: Supplementary file 1 [file Data_Sheet_1.docx]

Supplementary information for

Genetic analysis of *Citrobacter* sp.86 reveals involvement of corrinoids in chlordecone and lindane biotransformation

Agnès Barbance^a,1^, Oriane Della-Negra^a,1^, Sébastien Chaussonnerie^1^, Valérie Delmas^1^, Delphine Muselet^1^, Edgardo Ugarte^1^, Pierre-Loïc Saaidi^1^, Jean Weissenbach^1^, Cécile Fischer^1^, Denis Le Paslier^*,1^ and Nuria Fonknechten^*,1,2^

^1^Génomique Métabolique, Genoscope, Institut François Jacob, CEA, CNRS, Univ Evry, Université Paris-Saclay, 91057 Evry, France

^2^Present address: Laboratoire de Cancérologie Expérimentale, IRCM, Institut François Jacob, CEA, Université Paris-Saclay, 92265 Fontenay aux Roses, France

^a^Co-first authors

***Correspondence:**Corresponding Author
Nuria Fonknechten

nuria.fonknechten@cea.fr

Denis Le Paslier

denis@genoscope.cns.fr

# Supplementary figures

Figure S1: A- Known chlordecone microbial transformation pathway in anaerobiosis (Chevallier et al., 2019). B- Known lindane microbial transformation pathway in anaerobiosis (Zhang et al., 2020).


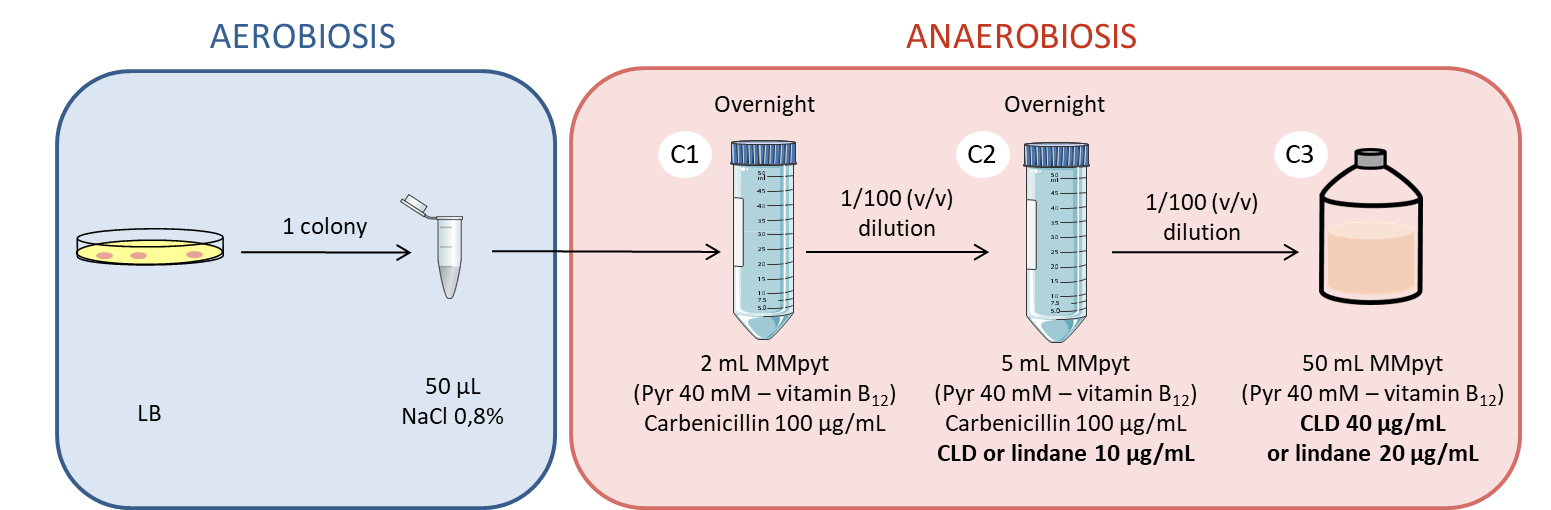


Figure S2: Schematic representation of the successive steps leading to anoxic microbial incubation of *Citrobacter* sp.86 and the knock-out mutant strains, for chlordecone or lindane biotransformation analysis (See Materials and Methods section).


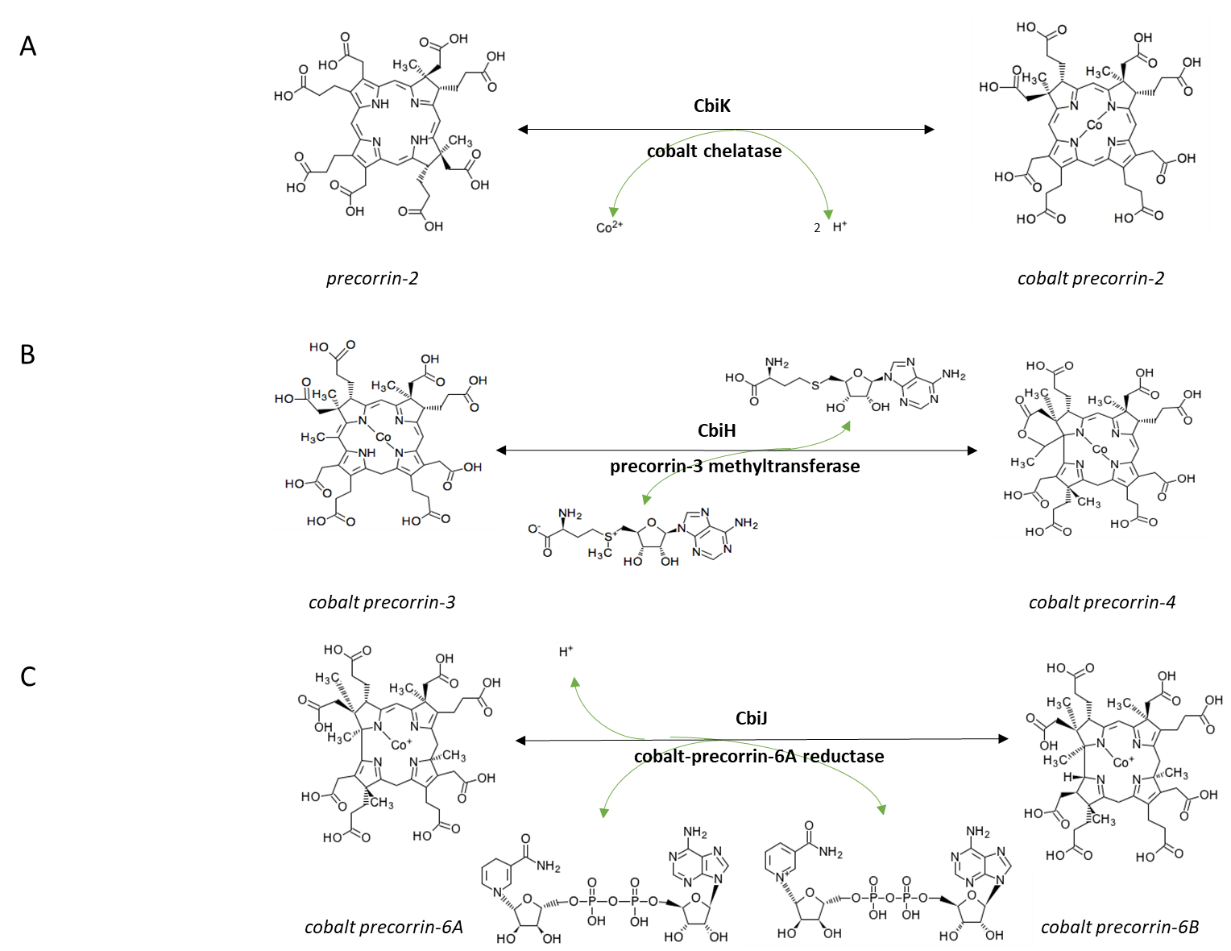

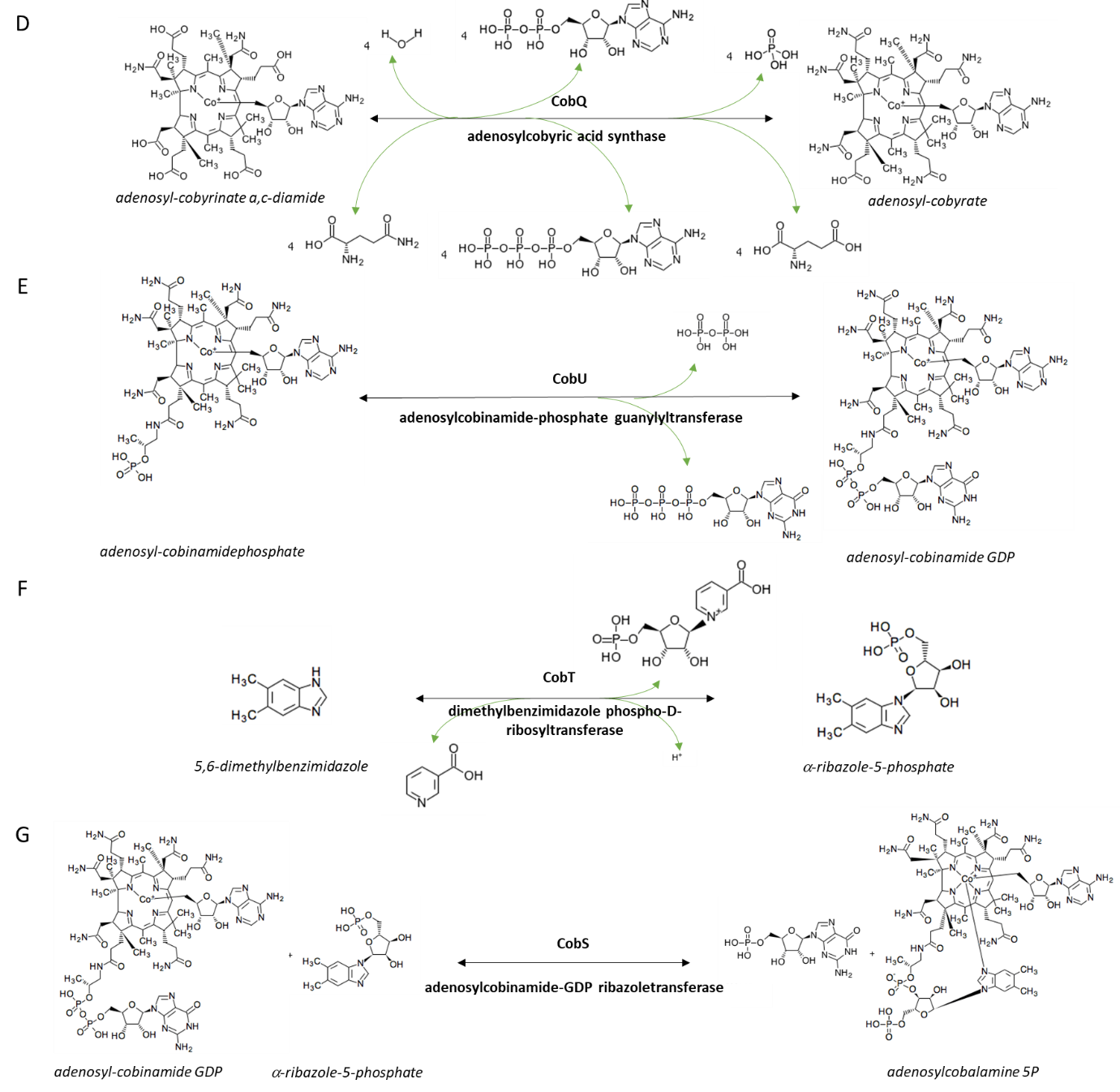


Figure S3: A-G - Details of selected enzymatic hypothetical reactions in the cobalamin biosynthesis pathway (according to the KEGG metabolic database).


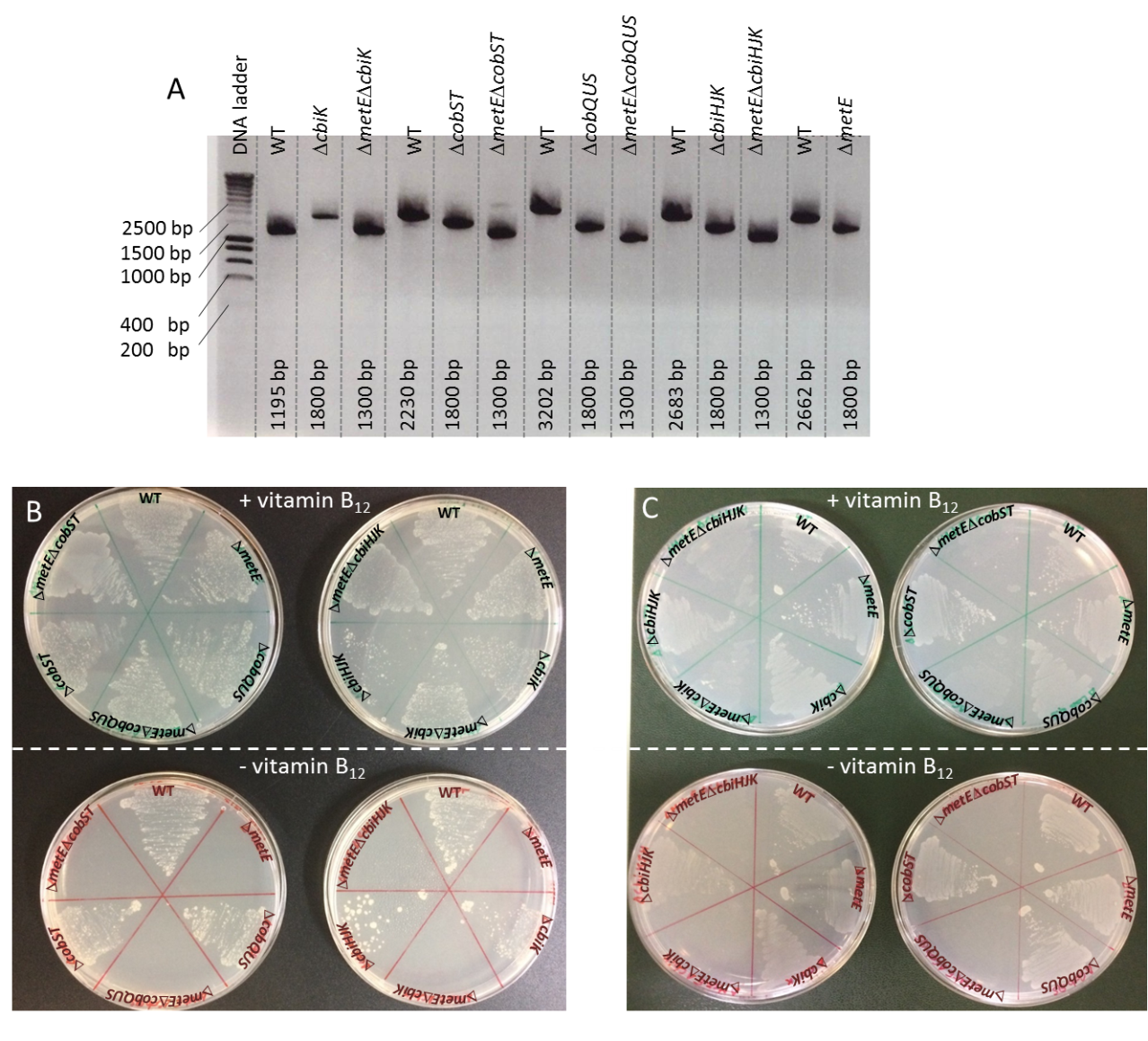


Figure S4: Phenotype-genotype concordance of the *Citrobacter* sp.86 mutant strains. A- PCR verification of genotypes; left lane: DNA ladder (Smart ladder, Invitrogen; expected amplicons size (in bp) from wild type and mutant strains are indicated at the bottom. For the wild type strain, the size corresponds to the length of the targeted region plus flanking regions (2 x 200 bp; see Materials and Methods section). For the mutant strains, the sizes correspond to the antibiotic cassette length plus 2 x 200 bp. In simple mutant strains, the deleted region was replaced by a kanamycin cassette (1,400 bp); in double mutant strains the deleted *cob/cbi* region was replaced by a chloramphenicol cassette (900 bp). B- Aerobic growth of wild-type and mutant strains of *Citrobacter* sp.86 on solid mineral medium. C- Anaerobic growth of wild-type and mutant strains of *Citrobacter* sp.86 on semi-solid mineral medium (see Materials and Methods section).


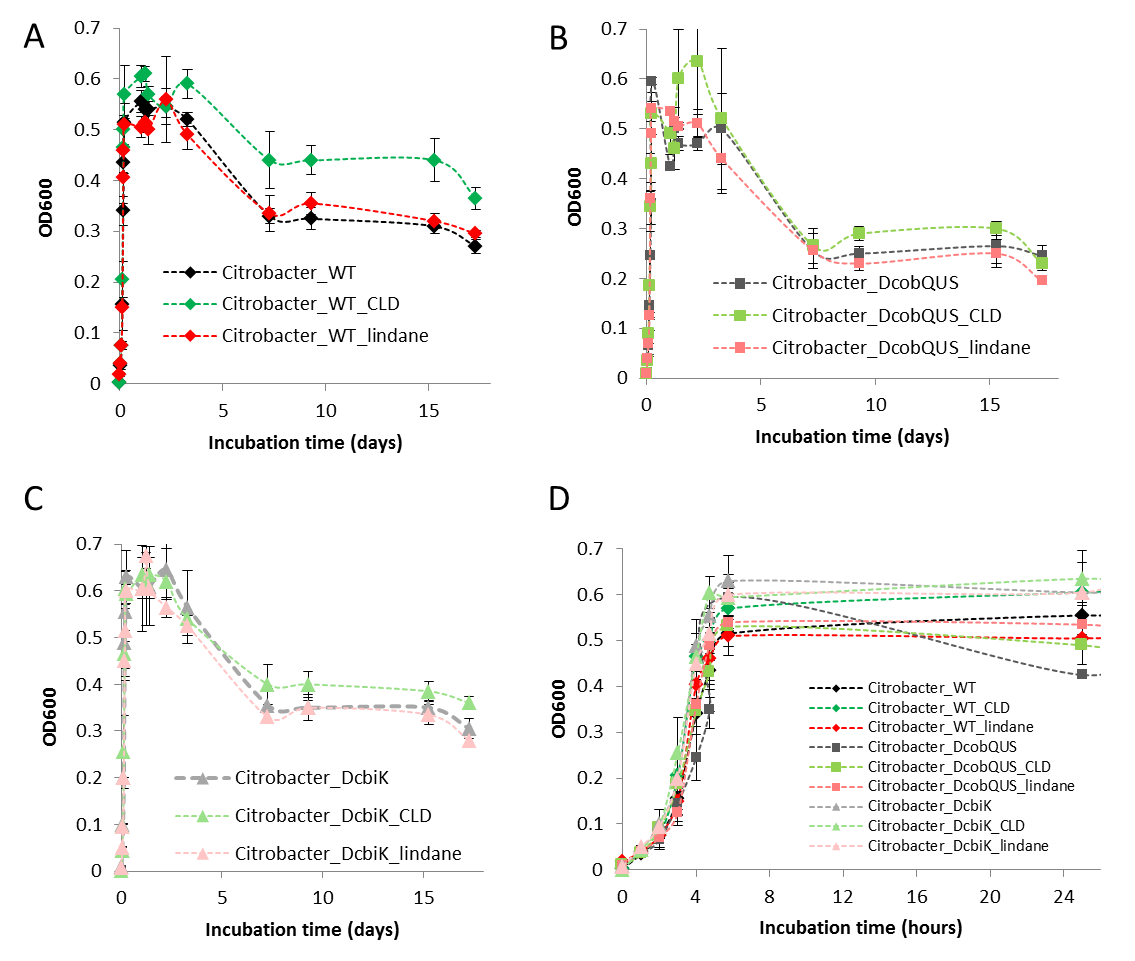


Figure S5: Growth curves of *Citrobacter* sp.86 and selected mutants, in MMpyt medium, supplemented or not with chlordecone or lindane (see Material and Methods section) and monitored by optical density (600 nm). A- Growth curves of *Citrobacter* sp.86 wild type for 17 days supplemented or not with chlordecone or lindane. B- Growth curves of *Citrobacter* sp.86 Δ*cobQUS* for 17 days supplemented or not with chlordecone or lindane. C- Growth curves of *Citrobacter* sp.86 Δ*cbiK* for 17 days supplemented or not with chlordecone or lindane. D- Growth curves of *Citrobacter* sp.86 and selected mutants, supplemented or not with chlordecone or lindane within the 24 first hours. OD_600_ = optical density at 600 nm. Numeric data are available in supplementary Xcel file.


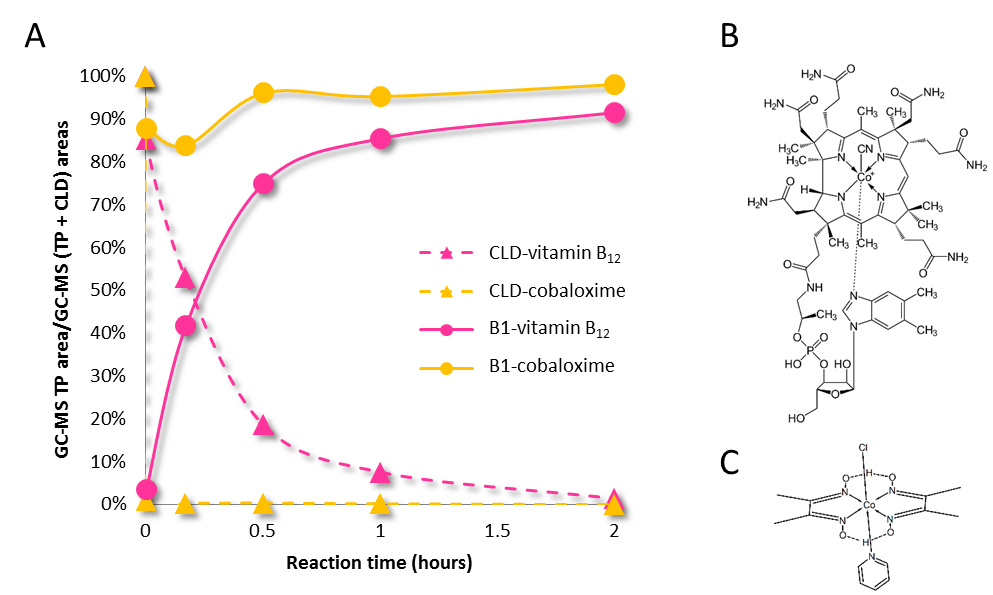


Figure S6: A- GC-MS monitoring of chlordecone chemical transformation and B1 appearance with vitamin B_12_ or chloro(pyridine)cobaloxime (abbreviated as cobaloxime). Y-axis is indicative and represents GC-MS peak area of chlordecone or B1 / the sum of chlordecone and B1 GC-MS peak areas. B- Structure of vitamin B_12_. C - Structure of chloro(pyridine)cobaloxime.

# Supplementary tables

| **Primer name** | **Sequence (5'->3')** |
| --- | --- |
| MetE_F50_Kan | TTCCCTGAATTAAAAATCCATAGGATTTACATATAATTAGAGGAAGTAAA**TGAGCGATCTGGAGCTGCTTC** |
| MetE_R50_Kan | CCCCCGACAACAGGCCCGGTGGCGACTTCGCCTTACCGGGCCCAGGAAGA**CATATGAATATCCTCCTTAG** |
| CobQUS_F50_Kan | GAAGATGAATTTTTCCGTTGTATGCGACAACGCGCAATCAAGGAGGCGTC**TGAGCGATCTGGAGCTGCTTC** |
| CobQUS_R50_Kan | GGAGTAAAGAGGTTAACGTTTGCATATGCGTTCTCATTGTGTTGGCTGAC**CATATGAATATCCTCCTTAG** |
| CobST_F50_Kan | GCGGCGGATGAGGTCTGGCTGGTGGTATCTGGTATTGGAGTCAAAATTAA**TGAGCGATCTGGAGCTGCTTC** |
| CobST_R50_Kan | CACGCTGACCAGCAAACTGCGCGGTTTACCGGCCAGGCAAGGTCACTTTA**CATATGAATATCCTCCTTAG** |
| CbiHJK_F50_Kan | GAAACCTTGCGTGAGCAGGGCGTCACTATCACTTTGGGAGTTTCACACTG**TGAGCGATCTGGAGCTGCTTC** |
| CbiHJK_R50_Kan | AATCAGATCGGCTGCTCCCGGGCCAGTGCTCAGGGCGTACAGTTTGCCGC**CATATGAATATCCTCCTTAG** |
| CbiK-F50_Kan | TTTGCGCAACGTTTGGCGCGCTGGCTGGCCGCTGCTTAAGGAATCAGAAA**TGAGCGATCTGGAGCTGCTTC** |
| CbiK-R50_Kan | AATCAGATCGGCTGCTCCCGGGCCAGTGCTCAGGGCGTACAGTTTGCCGC**CATATGAATATCCTCCTTAG** |

Table S1: PCR primers used to create the knock-out strains of *Citrobacter* sp.86. These primers include sequences (written in bold) that can amplify both the kanamycin and the chloramphenicol cassette. The upstream sequence is specific of the *Citrobacter* sp.86 targeted genomic region.

| **Primer name** | **Sequence (5'->3')** |
| --- | --- |
| MetE_verExt1-f | TCGGCATGAACAAATTGCGC |
| MetE_verExt1-r | AGGAGAGCGTGGAGACCATG |
| CobQUS_verExt1-f | TACGTCATGGCGAAGTGCTG |
| CobQUS_verExt1-r | ACACGCGGTACACCGTGTAG |
| CobST_verExt1-f | ATAGCTGGGATTACGCCGC |
| CobST_verExt1-r | GAACGGTAATCGTCAGCGG |
| CobST_verExt2-f | GGAGATCCAGACGCTGATCG |
| CobST_verExt2-r | TTTCCAACCAGACGACTTCC |
| CbiHJK_verExt1-f | TGATCCAGCTTGCCTCCTGC |
| CbiHJK_verExt1-r | GCGGTCACTTCATCCCAGAC |
| CbiK_verExt1_f | ATCGTCAGTGTCAGGCTGAC |
| CbiK_verExt1_r | CTGTGTTCGTGCCGATGTAC |

Table S2: PCR primers used to verify the knock-out strains of *Citrobacter* sp.86.

| **Plasmid name** | **Usage** | **Reference** |
| --- | --- | --- |
| pKD3 | Chloramphenicol resistance gene amplification | Datsenko et Wanner 2000 |
| pKD4 | Kanamycin resistance gene amplification | Datsenko et Wanner 2000 |
| pKD46-Gm | Contains the Red recombinase gene | Doublet et al. 2008 |

Table S3: Plasmids used in the study

| **Strain name** | **Genotype** | **Reference** |
| --- | --- | --- |
| *Citrobacter* sp.86 | Wild type | (Chaussonnerie et al. 2016) |
| Δ*cobQUS* | *Citrobacter* sp.86 Δ(*cob*Q *cobU* *cob*S)::*kan* | This work |
| Δ*cobST* | *Citrobacter* sp.86 Δ(*cob*S *cob*T)::*kan* | This work |
| Δ*cbiHJK* | Citrobacter sp.86 Δ(*cbi*H *cbi*J *cbi*K)::*kan* | This work |
| Δ*cbiK* | *Citrobacter* sp.86 Δ*cbiK*::*kan* | This work |
| Δ*metE* | *Citrobacter* sp.86 Δ*met*E::*kan* | This work |
| Δ*metE* Δ*cobQUS* | *Citrobacter* sp.86 Δ*met*E::*kan* Δ(*cob*Q *cob*S)::*cat* | This work |
| Δ*metE* Δ*cobST* | *Citrobacter* sp.86 Δ*met*E::*kan* Δ(*cob*S *cob*T)::*cat* | This work |
| Δ*metE* Δ*cbiHJK* | *Citrobacter* sp.86 Δ*met*E::*kan* Δ(*cbi*H *cbi*J *cbi*K)::*cat* | This work |
| Δ*metE* Δ*cbiK* | *Citrobacter* sp.86 Δ*met*E::*kan* Δ*cbi*K::*cat* | This work |

Table S4: *Citrobacter* sp.86 strains used in this study.


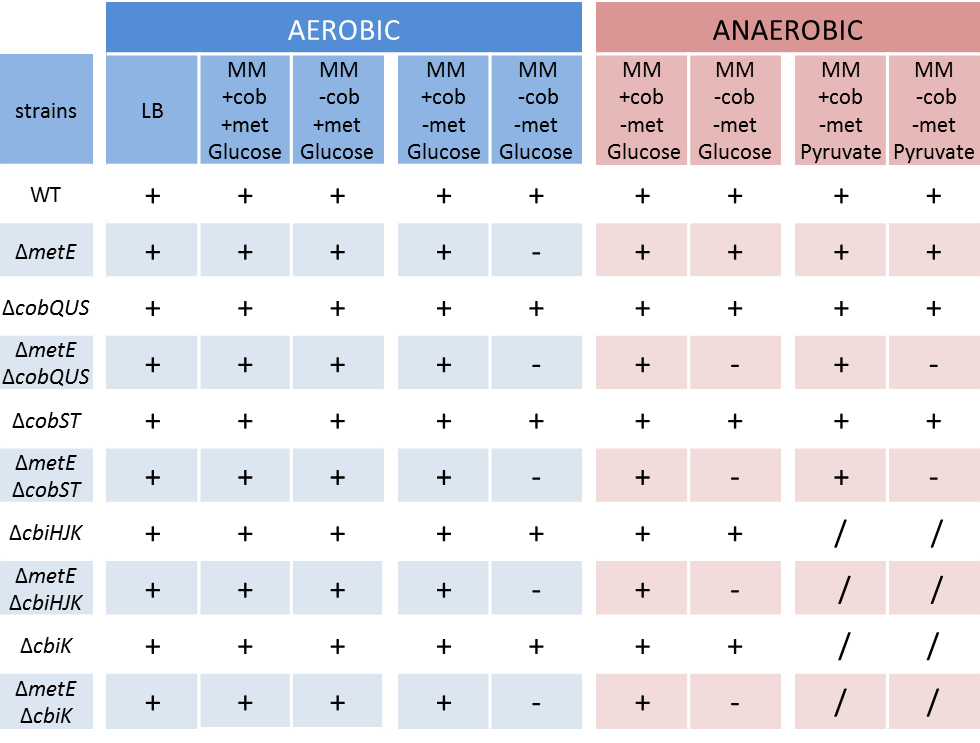


Table S5: Growth on solid medium of wild type and cobalamin-biosynthesis impaired mutant strains of *Citrobacter* sp.86 under aerobic and anaerobic conditions. After growth on rich medium LB, the strains were streaked on mineral medium (MM) supplemented with glucose (20 mM) or pyruvate (40 mM). The presence/absence of cobalamin and methionine were indicated by cob/met (+/-). (+) indicates growth, (-) absence of growth. (/): not tested on pyruvate as carbon source.
